# Perception about benefits and risks related to combined hormonal contraceptives use in women with Lynch syndrome

**DOI:** 10.1080/07853890.2024.2370568

**Published:** 2024-06-26

**Authors:** Fabio Barra, Umberto Perrone, Simone Ferrero, Stefano Bogliolo, Silvia Ottonello, Claudio Gustavino, Angela Iasci, Giovanni Grandi, Alessandra Pulliero, Maria Grazia Centurioni, Alberto Izzotti

**Affiliations:** a Unit of Obstetrics and Gynecology, P.O. ‘Ospedale del Tigullio’ – ASL4, Chiavari, GE, Italy; b Department of Health Sciences (DISSAL), University of Genoa, Genoa, Italy; c Department of Neurosciences, Rehabilitation, Ophthalmology, Genetics, Maternal and Child Health (DiNOGMI), University of Genoa, Genoa, Italy; d Academic Unit of Obstetrics and Gynecology, IRCCS Ospedale Policlinico San Martino, Genoa, Italy; e Unit of Obstetrics and Gynecology, IRCCS Ospedale Policlinico San Martino, Genoa, Italy; f Department of Medical and Surgical Sciences for Mother, Child and Adult, University of Modena and Reggio Emilia, Azienda Ospedaliero-Universitaria di Modena, Modena, Italy; g Unit of Mutagenesis and Cancer Prevention, IRCCS Ospedale Policlinico San Martino, Genoa, Italy; h Department of Experimental Medicine (DIMES), University of Genoa, Genoa, Italy

**Keywords:** Lynch syndrome, combined hormonal contraceptive, hormones, colorectal cancer, breast cancer, ovarian cancer, endometrial cancer, contraception, dysmenorrhea

## Abstract

**Objective:**

Lynch syndrome (LS) is a hereditary condition associated with an increased risk of colorectal and endometrial cancer. This study aimed to assess the knowledge, attitudes, and beliefs of women with LS regarding combined hormonal contraceptive (CHC) use compared to a control group of healthy women.

**Methods:**

Pre-menopausal women with LS (*n* = 43) and an age-matched control group of healthy women (*n* = 128) participated in this prospective, cross-sectional study (NCT05909410). Participants completed an electronic questionnaire evaluating perceptions of CHC use and its impact on various cancers, medical conditions, and symptoms. Statistical analysis compared responses between the two groups, with reported *p*-values.

**Results:**

Women with LS were less likely to use CHCs compared to the control group (*p* = 0.03) and had a more negative perception of CHCs’ impact on colorectal cancer (*p* = 0.023) and endometrial cancer (*p* = 0.028). Limited knowledge was observed in both groups regarding the protective effects of CHCs against colorectal and ovarian cancer. Perceptions of CHC use and its impact on symptoms and chronic diseases did not significantly differ between the groups (*p* > 0.05). CHC use was not associated with greater awareness of the protective effect against colorectal (*p* = 0.89) and endometrial cancer (*p* = 0.47), but it was associated with a desire for contraception (OR 21.25; 95% CI 1.16 to 388.21; *p* = 0.039).

**Conclusion:**

This study highlights contrasting perceptions of CHCs and their implications in oncology between women with LS and healthy women. Tailored counselling and support strategies are crucial for empowering women with LS to make informed decisions about their gynaecologic health.

## KEY MESSAGES

- This study illuminates divergent perceptions of combined hormonal contraceptives and their oncological implications between women with Lynch syndrome and healthy women.
- Tailored counseling and supportive strategies are essential for empowering women with Lynch syndrome to make informed decisions regarding their gynecologic health.

## Introduction

Lynch syndrome (LS) is a genetic condition characterized by an increased susceptibility to certain types of cancer, particularly colorectal cancer and other gastrointestinal cancers. This hereditary condition is caused by germline pathogenic variants in DNA mismatch repair (MMR) genes, including MLH1, MSH2, MSH6, and PMS2, as well as EPCAM. While colorectal cancer (cumulative lifetime risks [CLR]: 10.4–57.1%) is the most commonly associated cancer in LS, women with this condition also have a significantly higher lifetime risk of developing endometrial cancer compared to the general population (CLR: 12.8–48.9%); additionally, these patients face an increased risk of ovarian cancer (CLR: 3.0–17.4%) [1].

Currently, managing cancer life-risks in patients with LS poses challenges. The Manchester International Consensus Group suggests annual surveillance for asymptomatic women from age 25, discussing symptoms and contraceptive needs [2]. Prophylactic hysterectomy with bilateral salpingo-oophorectomy, recommended from ages 35–40 after childbearing, aims to detect and manage potential abnormalities early [3]. Nevertheless, in the context of LS, women’s preferences for gynaecologic cancer risk management options, including the type and frequency of interventions, have been relatively understudied [4].

It is known that use of combined hormonal contraceptives (CHCs) in premenopausal women plays not only the role of preventing unintended pregnancies; the physiological actions of the estrogens and progestins also provide important non-contraceptive benefits, including treatment of common gynaecological and non-gynaecological medical conditions [5]. Some data suggested that long-term exposure to CHC has been associated with a reduced risk of endometrial cancer in the general population [6], and also, in LS [7,8]. Others demonstrated that CHC use may be associated with a reduced risk of colorectal cancer [9,10], although the potential impact of CHCs on colorectal cancer risk in women with LS remains to be clarified. Also considering the benefits on specific cancer risks, the Manchester International Consensus Group recommends the use of CHCs for women seeking contraception [2]. Differently, the National Comprehensive Cancer Network (NCCN) guidelines comment on contraceptive use for women with LS [11], they do not formally recommend its use.

Nevertheless, in daily practice, myths and taboos regarding side effects and long-term consequences of CHC use on women’s health need to be fully addressed also in the general population [12]. The awareness of the effects of hormonal therapies in women at high risk of developing endometrial cancer, colorectal, or ovarian cancer, such as those affected by LS, is even more limited, with few published studies addressing the perception about benefits and risks related to CHCs only in BRCA pathogenic variant carriers [13,14]. Understanding women’s preferences and their unique perspectives is crucial for tailoring effective counselling and support strategies [15]. Accurate information regarding the oncological risks associated with CHC is essential for facilitating shared decision-making between women and their healthcare providers in this specific population. Understanding women’s preferences and their unique perspectives is crucial for tailoring effective counselling and support strategies [15]. Therefore, this prospective study aims to evaluate the knowledge, attitudes, and beliefs of women with LS concerning CHCs and their potential effects on specific disease development and cancer risk, comparing them to the general population.

## Materials and methods

This prospective, cross-sectional, observational study aimed to assess the knowledge, attitudes, and beliefs of women (individuals assigned female at birth) with LS regarding the use of CHCs and their potential effects on disease development and cancer risk. The study was conducted from September 2021 to May 2023.

Consecutive pre-menopausal healthy women with confirmed diagnosis of LS were included in the study group, being enrolled at the IRCCS San Martino University Hospital (Genoa, Italy), the regional referral centre for hereditary cancers. The diagnosis of LS was based on established diagnostic criteria, including genetic testing for germline pathogenic variants in DNA mismatch repair (MMR) genes (MLH1, MSH2, MSH6, and PMS2) and EPCAM. Additionally, an immunochemistry panel for MMR proteins was performed.

A control group was identified, comprising, age-matched pre-menopausal healthy women without a previous diagnosis of LS. These individuals were referred for a routine gynaecological examination during the study period at the IRCCS San Martino University Hospital (Genoa, Italy) and at P.O. ‘Ospedale del Tigullio’ – ASL4 Liguria (Chiavari [GE], Italy).

Exclusion criteria for both groups included a prior history of oncological diseases, a personal history of endometrial cancer, colorectal cancer, ovarian cancer, or breast cancer, as well as previous salpingectomy, salpingo-oophorectomy, or hysterectomy (any route). This also encompassed all risk-reducing surgical procedures typically performed for women with LS.

### Data collection

During routine gynaecological assessments, comprehensive clinical information was collected from all participants. Data included age, parity, total number of vaginal or cesarean deliveries, history of abortion, and prior abdominal and gynaecological surgeries. To collect data on the participants’ perceptions and opinions, an electronic questionnaire was administered *via* mailing-list to all enrolled participants, both in the LS and control groups. The questionnaire, previously utilized in other recent Italian studies involving premenopausal women with BRCA pathogenic variants [13], addressed the use of CHCs and their potential impact on: 1) specific types of cancer (breast cancer, epithelial ovarian cancer, endometrial cancer, cervical cancer, colorectal cancer, lymphoma); 2) medical pathological conditions (venous thrombosis, breast cysts, cardiovascular incidents, anaemia, fetal abnormalities, infertility, ectopic pregnancy, sexually transmitted infections); 3) symptoms (headache, weight gain, reduction in sexual desire, vaginal dryness, increased/decreased appetite, mood swings, depressive mood, abnormal uterine bleeding, dysmenorrhea, acne). Participants used a Likert scale ranging from −5 to +5 to assess their perception of the impact of CHCs on these variables. The scale allowed participants to rate the degree of reduced risk to increased risk for cancers and medical conditions, as well as improvement to worsening for symptoms: specifically, a score of −5 indicated a perception of reduced risk or improvement in the given outcome, while a score of +5 indicated a perception of increased risk or worsening of the outcome. Scores close to 0 denoted a neutral or no perceived effect.

### Ethics approval

Specific informed consent was obtained from each participant for the use of sensitive data for scientific purposes. This study was approved by the local ethics committee (CERLIGURIA: CE2386PR080623-13249). Women participating in the study provided written informed consent. This study was registered in Clinicaltrial.gov (NCT05909410). This study followed the STROBE checklist for case-control study (Supplementary File 1) [16].

### Statistical analysis

The sample size was calculated considering the number of women with LS who could have been potentially enrolled in the IRCCS San Martino University Hospital (Genoa, Italy). Assuming a pooled SD of 1 unit and a 1:3 ratio, the study would require at least a sample size of 42 women with LS and 126 healthy women to achieve a power of 80% and a level of significance of 5% (two-sided) for detecting a true difference in means of 0.5 units in Likert scale values between the groups.

The electronic questionnaire was built by using Google Forms (Google LLC, Mountain View, California, United States), a dedicated web software (Google Forms, Mountain View, California, United States). The responses to the questionnaires from women with and without LS were analyzed and compared for investigating the perceptions in each group.

Categorical variables were expressed as frequency and percentage, while continuous data were reported as mean ± standard deviation (SD). Statistical comparisons between categorical variables were performed using either chi-square test or Fisher’s exact test, depending on the sample size and expected cell frequencies. Within-group comparisons were conducted using the *T*-test for paired data and the Mann-Whitney U test for normal and non-normal data distributions, respectively. To explore the factors associated with the current CHC use in women with LS, a multivariate logistic regression analysis was performed. Adjusted odds ratios (ORs) and 95% confidence intervals (CIs) were calculated to determine the strength and significance of the associations. Statistical analysis was performed using SPSS software version 26.0 (SPSS Science, Chicago, IL). Correlations were considered significant at *p*-values < 0.05.

## Results

Out of the initial pool of 186 women who were considered potentially eligible for the study, eight participants were subsequently excluded. The reasons for exclusion were incomplete questionnaire answers in 11 participants, and withdrawal of consent by 4 participants. The final analysis comprised 43 premenopausal women with LS (mean age: 38.2 ± 8.1) and 128 healthy women of similar age (39.6 ± 8.3 years; *p* = 0.13). The demographic characteristics of both study populations are reported in Table 1.

**Table 1.** Demographic characteristics and family history variables of study groups.

Demographic variable	Patients with Lynch syndrome (*n* = 43)	Healthy women (*n* = 128)	*P* value
Age, years (mean ± SD)	38.2 ± 8.1	39.6 ± 8.3	*p* = 0.33
Race/ethnicity (*n*, %)			*p* = 0.21
*White*	40 (93.0)	121 (94.5)	
*Black*	2 (4.7)	4 (3.1)	
*Hispanic*	1 (2.3)	3 (2.4)	
Pathogenic variants (*n*, %)			
*MLH1*	18 (41.9)	**-**	**-**
*MSH2*	19 (44.1)	**-**	**-**
*MSH6*	1 (2.3)	**-**	**-**
*PMS2*	3 (7.0)	**-**	**-**
*EPCAM*	2 (4.7)	**-**	
Education level (*n*, %)			*p* = 0.27
*Middle school*	4 (9.3)	11 (8.6)	
*High school*	21 (48.9)	60 (46.9)	
*University*	17 (39.5)	53 (41.4)	
*Other Higher*	1 (2.3)	4 (3.1)	
Marital status (*n*, %)			*p* = 0.34
*Single*	5 (11.6)	13 (10.2)	
*Married*	29 (67.4)	91 (71.1)	
*Divorced/widows*	9 (21.0)	23 (18.0)	
*Unknown*	–	1 (0.7)	**-**
Sexually active (*n*, %)	41 (95.3)	121 (94.5)	*p* = 0.87
Previous parity (*n*, %)	16 (37.2)	50 (39.1)	*p* = 0.83
Any oncological family history (*n*, %)			
*Endometrial cancer*	27 (62.8)	7 (5.5)	*p* < 0.001
*Ovarian cancer*	11 (25.6)	4 (3.1)	*p* = 0.003
*Colorectal cancer*	41 (95.3)	24 (18.8)	*p* < 0.001
*Other cancers*	16 (37.2)	43 (33.6)	*p* = 0.22
Concomitant gynaecological diseases at ultrasound (*n*, %)			
*Endometriosis*	4 (9.3)	19 (14.8)	*p* = 0.18
*Adenomyosis*	8 (18.6)	28 (21.9)	*p* = 0.57
*Uterine fibroids*	11 (25.6)	27 (21.1)	*p* = 0.73
*Uterine malformations*	–	1 (0.7)	**-**
*Benign ovarian cyst*	5 (11.6)	18 (14.1)	*p* = 0.29

A lower proportion of women with LS were currently using (13.9%) or had used before (18.6%) CHCs at the time of the study compared to healthy women (31.3% and 43.8%, respectively; *p* = 0.03 and *p* = 0.003). Furthermore, women with LS had a significantly shorter mean current CHC duration (24.0 months vs 36.2 months, *p* = 0.02) and total CHC duration (27.6 months vs 49.6 months, *p* = 0.01; Table 2).

**Table 2.** Characteristics of combined hormonal contraceptives in the study groups.

Variable	Patients with Lynch syndrome (*n* = 43)	Healthy women (*n* = 128)	*P* value
Present users (*n*, %)	6 (14.0)	40 (31.2)	0.03
*Pill*	5 (11.6)	31 (24.2)	
*Vaginal ring*	1 (2.3)	7 (5.5)	
*Patch*	–	2 (1.7)	
Current duration use, months (median IQR 1–4)	21.0 (15.5–32.0)	41.0 (27.75–82.0)	0.02
Past users	8 (18.6)	56 (43.8)	0.003
*Pill*	7 (16.3)	48 (37.5)	
*Vaginal ring*	–	7 (5.5)	
*Patch*	1 (2.3)	1 (0.8)	
Past duration use, months (median IQR 1–4)	29.5 (12.8–53.0)	36.0 (22.8–96.0)	0.24
No previous use (*n*, %)	30 (69.8)	51 (39.8)	0.03
Total duration of use, months (median IQR 1–4)	25.0 (16.0–53.0)	36.2 (31.0–123.0)	0.01

**Figure 1.**
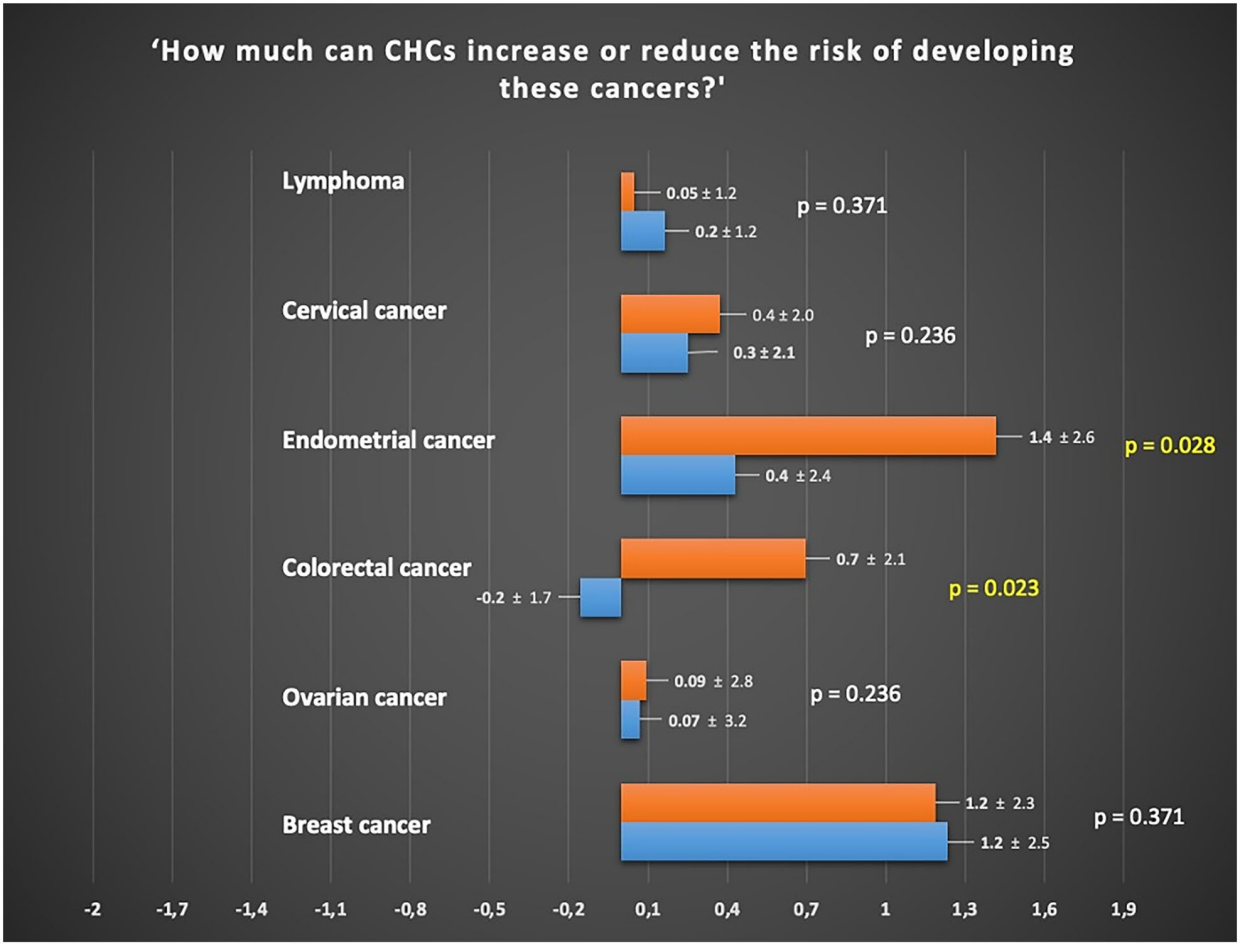
Perception of women with Lynch syndrome (orange) and healthy women (blue) regarding the association between combined hormonal contraceptives and cancers, assessed using a Likert scale ranging from −5 to +5. For a specific cancer, a score of −5 indicates the strongest perception of reduced risk, while a score of 0 indicates a neutral perception of neither reduced nor increased risk, and a score of +5 indicates the strongest perception of increased risk.

The protective effects of CHCs against colorectal and ovarian cancer were similarly unknown by women with LS and healthy women (0.7 ± 2.1 and −0.2 ± 1.7 points, respectively, for colorectal cancer; 0.09 ± 2.8 and 0.07 ± 3.2 points, respectively, for ovarian cancer). However, the first group of women held a negative perception regarding the impact of CHCs on the onset of colorectal cancer, which differed from the neutral perception expressed by healthy women (*p* = 0.023). In both groups, there was a negative perception related to the use of CHCs and the risk of developing endometrial cancer (1.4 ± 2.6 and 0.4 ± 2.4), although women with LS held a more negative perception on this risk (*p* = 0.028). Both groups had a negative perception without inter-group differences on the role of CHCs in the risk of developing breast cancer (1.2 ± 2.3 and 1.2 ± 2.5; *p* = 0.371). Lastly, a not different negative impact of CHCs on the risk of cervical cancer onset was reported by women with LS and healthy women (0.4 ± 2.0 and 0.3 ± 2.1; *p* = 0.236). Figure 1 reports all the specific perceptions of both groups concerning the relationship between CHCs and cancer.

**Figure 2.**
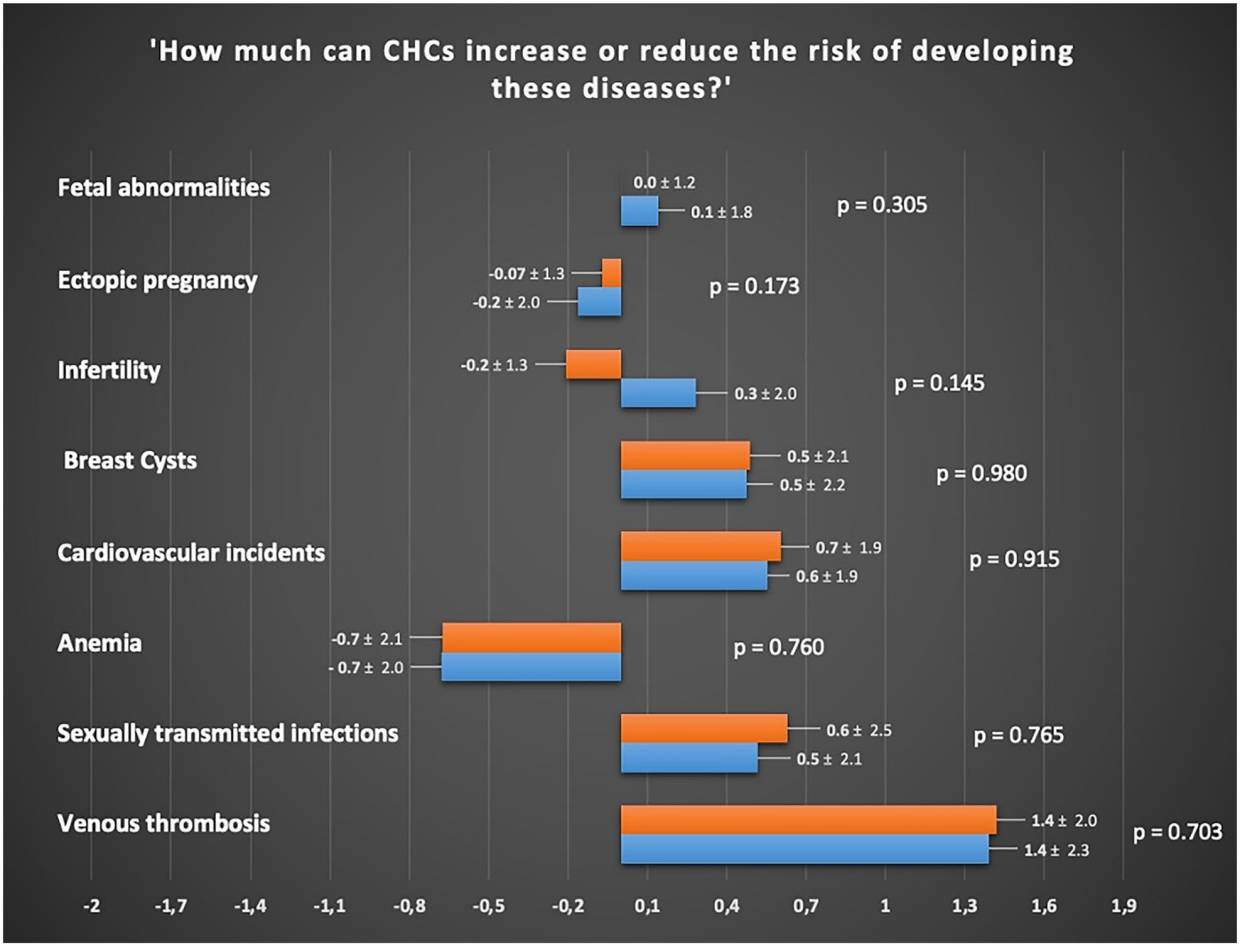
Perception of women with Lynch syndrome (orange) and healthy women (blue) regarding the association between combined hormonal contraceptives and medical diseases, assessed using a Likert scale ranging from −5 to +5. For a specific medical disease, a score of −5 indicates the strongest perception of reduced risk, while a score of 0 indicates a neutral perception of neither reduced nor increased risk, and a score of +5 indicates the strongest perception of increased risk.

**Figure 3.**
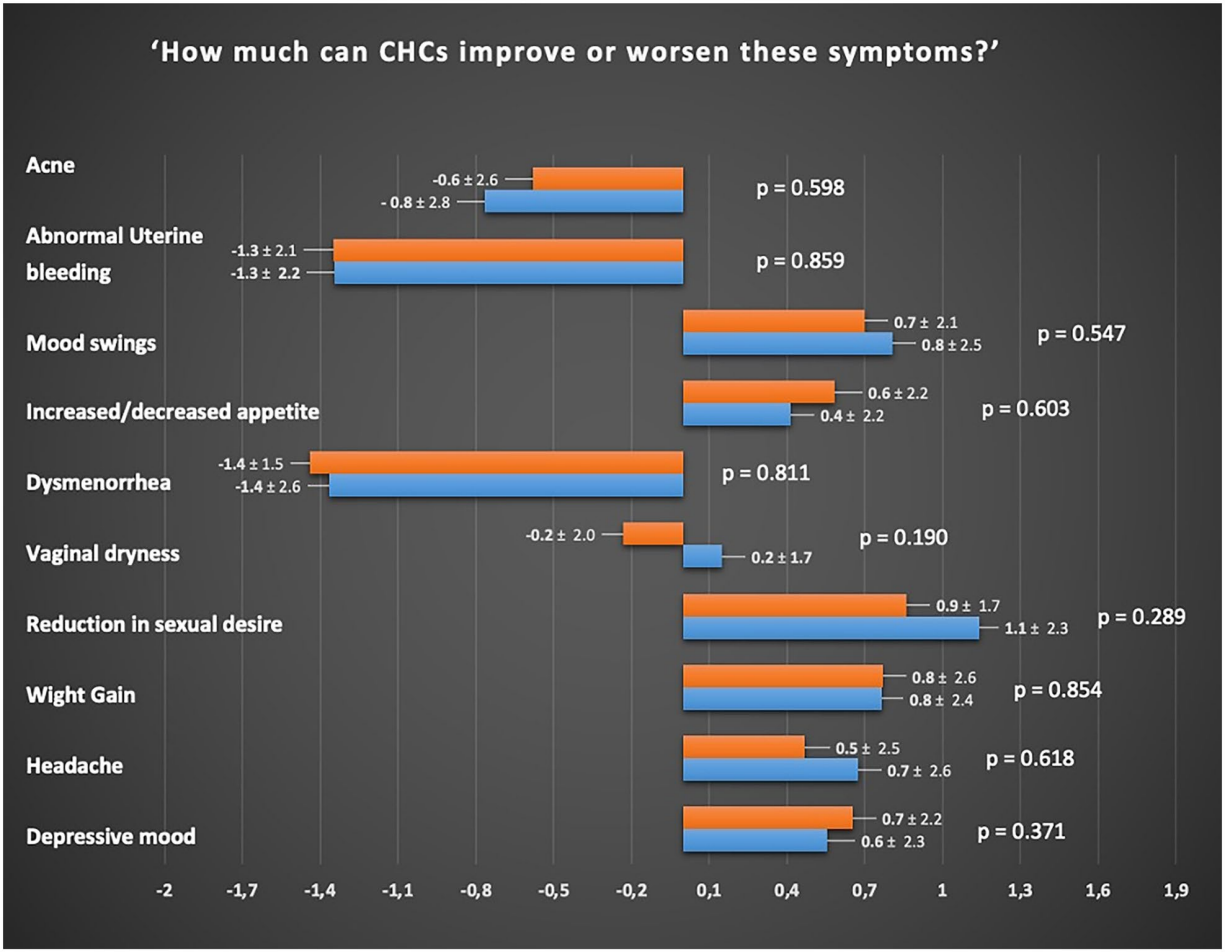
Perception of women with Lynch syndrome (orange) and healthy women (blue) regarding the relationship between combined hormonal contraceptives and symptoms, evaluated using a Likert scale ranging from −5 to +5. For a specific symptom, a score of −5 indicates the strongest perception of improvement, while a score of 0 indicates a neutral perception of neither improvement nor worsening, and a score of +5 indicates the strongest perception of worsening.

Perceptions regarding the effects of CHC use on the risk of developing adverse symptoms and medical diseases did not differ significantly between the two groups (all the *p* > 0.05). Figures 2 and 3 report the specific perception of both groups concerning the relationship between CHC use and development of symptoms and chronic disease, respectively.

In women with LS, CHC use at the time of the study was not associated with awareness of the protective effect against colorectal (*p* = 0.89) and endometrial cancer (*p* = 0.47); however, it was associated with the desire for contraception (OR 21.25; 95% CI 1.16 to 388.21; *p* = 0.039). The variables associated with CHC use in this group of women are presented in Table 3.

**Table 3.** Multivariate logistic regression analysis of independent variables associated to the current CHC use in patients with lynch syndrome.

Independent variable	Coefficient (B)	Wald *p*-value	OR	95%CI	*P* value
*Gynecological cancers*					
Negative impact on breast cancer risk	−2.07	2.68	0.13	0.11–1.50	0.10
Positive impact on ovarian cancer risk	1.23	1.32	3.41	0.42–27.63	0.25
Positive impact on colorectal cancer risk	0.18	0.02	1.20	0.08–22.33	0.89
Positive impact on endometrial cancer risk	0.84	0.52	2.31	0.24–22.33	0.47
Negative impact on cervical risk	1.77	5.72	5.87	0.56–61.80	0.14
*Need and symptoms*					
Menstrual cycle irregularity	0.18	0.13	1.20	0.51–28.3	0.91
Dysmenorrhea	3.02	3.54	20.47	0.88–474.29	0.06
Acne or other hyperandrogenism signs	1.75	0.45	5.76	0.03–974.21	0.50
Need for contraception	3.06	4.25	21.25	1.16–388.21	0.039

## Discussion

This study aimed to assess the knowledge, attitudes, and beliefs of women with LS regarding the use of CHCs and their potential effects on disease development and cancer risk, comparing them to a control group of age-matched healthy women. The results underscore the importance of implementing counselling and support strategies for women with LS, particularly concerning gynaecologic cancer risk management.

Our findings indicate that women with LS were less likely to have used or be currently using CHCs compared to healthy women (Table 2). This could be attributed to a heightened awareness of their hereditary cancer risk. Indeed, a previous study evaluated cancer worry perceived by women with LS: 59.6%, 37%, and 27.7% of them were characterized as having ‘a lot’ of worry about colorectal, endometrial cancer and ovarian cancer risks, respectively. By using a 0 to 100 scale (0 = no chance; 100 = definitely), the median perceived risks of developing colorectal, endometrial, and ovarian cancers by these women were 75%, 60%, and 30%, respectively [17]. The low use of CHC could be related to an unbalanced perception by women with LS concerning the potential impact of exogenous hormones on cancer development.

In the current literature, the use of CHCs has been associated with both protective and adverse effects on cancer risk, depending on the type of cancer [18]. Some data have suggested that CHC use may be associated with a reduced risk of colorectal cancer [9, 10], although the potential impact of CHCs on colorectal cancer risk in women with LS has not been specifically investigated. A notable finding of our study was the negative perception held by women with LS on the impact of CHCs on colorectal cancer onset. This perception differed significantly from the neutral opinion expressed by healthy women. This negative perception reinforces the significance of colorectal cancer as a major concern for in patients with LS [17]. In the future, epidemiological investigations regarding the use of CHCs and their association with colorectal cancer risks in this setting will be crucial for developing high-quality information and education strategies, especially if their beneficial effect as a long-term chemoprotective strategy will be demonstrated.

CHCs have been found to potentially increase the risk of breast cancer [19,20], which does not seem to be enhanced in women with LS in comparison to the general population [21]. Concerning this point, both women with LS and healthy women attributed a negative perception on CHCs on the onset of breast cancer with no inter-group difference; nevertheless, it cannot be excluded that both groups overestimated this cancer risk, which amounts just to 1.3 (95% CI 1.0–1.6) extra cases per 10,000 person-years in comparison to women who have never used CHCs before [20].

According to our results, the protective effects of CHCs against ovarian are similarly unknown between women with LS and healthy women. A previous systematic review with meta-analysis reported a significant reduction in ovarian cancer incidence in ever-CHC users compared with never-users (OR 0.73), with a significant duration-response relationship and a reduction in the incidence of more than 50% among women using them for 10 or more years [22]; however, at the best of our knowledge, no specific studies have evaluated the impact of CHCs on reducing this ovarian cancer in LS.

CHC use has been associated with a reduced risk of endometrial cancer in the general population [6]. Notably, an observational study showed that CHC use is associated with lower risk for endometrial cancer in women with LS (HR 0.39; 95% CI 0.23–0.64; *p* < 0.001) [7]. Another small study demonstrated a decrease in endometrial epithelial proliferation as measured by Ki-67 positive cells and the presence of inactive and/or secretory-type glands after the short-term exposition to CHCs or progestins [8]. In our study, both healthy women and those with LS showed a wrongly negative perception related to the use of CHCs and the risk of developing endometrial cancer. These results confirmed the lack of awareness about the benefit of using CHCs on preventing endometrial cancer reported also in the general population by a previous study [12]. However, women with LS had a significantly more negative opinion on this matter compared to the control group. This data could be attributed to the increased general lifetime risk of endometrial cancer faced by women with LS; additionally, it may heighten women’s concerns regarding the potential adverse effects of CHCs [15,23], which, otherwise, could have also a chemoprotective role and even become a future endometrial preventive option for these women [2].

Lastly, both groups had a neutral and non-significant perception of the risk of developing lymphoma with CHC use. This is consistent with current literature, which indicates that CHCs are unlikely to affect the risk of developing lymphoma [19].

In general, it is possible to assume that, as previously demonstrated in BRCA pathogenic variant carriers [13], women with LS may also have an altered perception of the impact of hormones in the pathogenesis of gynaecological cancers (Figure 1). On opposite, the potential protective effects of hormone-related factors on colorectal, endometrial, and ovarian cancers should be reassuring for women with LS. Therefore, the finding of the current study confirms that there is a need to improve awareness and understanding of the potential benefits of CHC use in both the general population and women with LS, particularly in the context of gynaecologic cancer risk management [9,24]. Healthcare providers should consider discussing the potential benefits and risks of CHC use with their patients, considering individual cancer risk profiles and personal preferences, to facilitate informed decision-making [25]. Overall, by improving knowledge and correcting misconceptions, educational interventions may help women with LS make informed decisions regarding CHC use.

Women with LS have similar perceptions to the general population regarding the effects of CHC use on the development of symptoms, including commonly experienced side effects during hormonal therapy (Figures 2 and 3) [24,26,27]. These findings suggest that counselling regarding the use of CHCs for symptom management in these women may not require additional emphasis beyond what is typically recommended for the general population [18]. Moreover, our analysis revealed that both groups demonstrate a comparable correct understanding of the non-contraceptive benefits of CHCs, such as the positive effects on dysmenorrhea and acne [28, 29] as well as the main adverse events related to their use, such as mood alteration and libido reduction [18,30].

This study had some strengths, including the presence of an age-matched control group without LS, the use of detailed electronic questionnaires, and the availability of a wide range of data from participants. The limitations of this study should be mentioned. The cross-sectional design of the study may introduce a potential bias, as the sample was not randomly selected. This could lead to an overrepresentation of participants with higher health awareness and greater motivation to cooperate with healthcare providers in clinical initiatives. Most of the recruitment of participants directly from a referral centre for hereditary cancer may have contributed to this bias. Furthermore, the Likert scale used in the questionnaires has a limited ability to compare perceived associations with the strength of known associations between CHC use and specific symptoms, diseases, and cancer risk. Notably, the questionnaire’s generic nature, previously employed by our research group [13], may not fully capture the nuances of all cancer risks associated with LS (e.g. pancreatic cancer [1]), potentially impacting the study’s ability to evaluate attitudes and beliefs related to CHC use. Moreover, the sample size calculation indicated that the study was powered to detect true differences in Likert scale values between the two groups, but it may not have been large enough to detect small differences (less than 0.5 points of difference). Lastly, most women with LS in this study harboured a pathogenic variant in MLH1 or MSH2 (*n* = 37; 86.0%). It would be valuable to explore potential differences in responses among women with pathogenic variants in different genes associated with LS. However, given the constraints of the sample size, we were unable to calculate associations. It is conceivable that perceptions may vary considerably among women whose risks are significant for certain cancers, such as endometrial and ovarian cancers (e.g. MSH2), compared to those with lower risk (e.g. PMS2) [1].

## Conclusion

This study emphasizes the necessity to enhance awareness and comprehension of the potential impacts of CHCs on specific disease development and cancer risk in women with LS. By considering clinical and psychosocial factors contributing to individual cancer risk perceptions, this research strives to contribute to informed decision-making, tailored counselling, and enhanced strategies for gynaecologic cancer risk management in LS women. This aims to promote shared decision-making and informed choices about their reproductive health and cancer risk management.

## Supplementary Material

Supplemental Material

## Data Availability

Data available on request from the authors.
